# The MAKS-s study: multicomponent non-pharmacological intervention for people with severe dementia in inpatient care – study protocol of a randomised controlled trial

**DOI:** 10.1186/s12877-020-01807-1

**Published:** 2020-10-15

**Authors:** Kristina Diehl, André Kratzer, Elmar Graessel

**Affiliations:** Centre for Health Services Research in Medicine, Department of Psychiatry and Psychotherapy, University Hospital Erlangen, Friedrich-Alexander University Erlangen-Nürnberg (FAU), Schwabachanlage 6, 91054 Erlangen, Germany

**Keywords:** Severe dementia, Psychosocial intervention, Behavioural and psychological symptoms of dementia, Quality of life, Nursing home, RCT

## Abstract

**Background:**

Nursing homes accommodate a large number of people with severe dementia. More than 80% of residents with dementia suffer from behavioural and psychological symptoms, that can have consequences on the perceived burden of the formal caregivers. Internationally, the number of studies on non-pharmacological interventions for people with severe dementia is very small. One way to reduce these symptoms is to meet the needs of people with severe dementia. The non-pharmacological group intervention MAKS-s, which we will investigate in this study, is intended to reduce the behavioural and psychological symptoms and to improve the quality of life of such people. Additionally, we will investigate the effects on the burden carried by formal caregivers.

**Methods:**

With the present study, we will investigate the effectiveness of a multicomponent non-pharmacological intervention for people with severe dementia living in nursing homes (primary target group). A power analysis indicated that 144 dementia participants should initially be included. In addition, a secondary target group (nursing home staff) will be examined with respect to their dementia-related stress experiences. The study will be conducted as a cluster randomised controlled trail in Germany with a 6-month intervention phase. The nursing homes in the waitlist control group will provide “care as usual.” The primary endpoints of the study will be the behavioural and psychological symptoms of dementia and the quality of life of people with severe dementia. The total duration of the study will be 18 months. Data will be collected by using observer rating scales.

**Discussion:**

The project has some outstanding quality features. The external validity is high, because it is situated in a naturalistic setting in nursing homes and is being carried out with available nursing employees. Due to this fact, a permanent implementation also seems to be possible. Since the participating nursing homes are disseminated across several German federal states and rural and urban regions, the results should be transferable to the entire population.

**Trial registration:**

ISRCTN15722923 (Registration date: 07 August 2019).

## Background

In Europe, more than one third of residents in nursing homes suffer from severe dementia [1–3]. A lack of meaningful occupation is often reported, especially for individuals with moderately severe and severe dementia in nursing homes [4]. Nevertheless, there are very few therapy options for this particularly vulnerable group of people. If at all, individual therapy is considered in relation to people with severe dementia (PWSDs). Guidelines for treating dementia and ecpecially for dealing with challenging behaviour in people with dementia have been published in several countries [5–7]. Only individual interventions for PWSDs are described there, a group intervention is missing from the list. The number of studies on interventions for PWSDs, especially those with high-quality study designs, is very low internationally [8]. This means that either no such approaches have been developed so far, or they have not been published internationally. To date, despite the finding that multicomponent interventions are more effective than single interventions [9], no studies have been conducted on a multicomponent group therapy adapted to the needs of PWSDs. In particular, there is no intervention concept with concrete goals and a structured manual for PWSDs.

The multicomponent MAKS intervention (standing for Motor stimulation, Activities of daily living stimulation, Cognitive stimulation, and Social functioning) was already scientifically approved in two RCTs [10, 11] for its effectiveness on psychological symptoms in people with mild to moderate dementia. We adapted this concept to address the needs of PWSDs. As Cohen-Mansfield and colleagues postulate, understanding unsatisfied needs and finding a remedy should be the starting point of any non-pharmacological intervention [12]. The “Unmet needs model” [12] assumes that dementia patients, due to their cognitive and motivational limitations, become less and less able to communicate and to meet their own needs. This leaves many needs unfulfilled. This frustration and the inability to express oneself verbally contributes to the frequently observed behavioural and psychological symptoms of dementia (BPSD) [12]. Symptoms such as apathy, depression, and anxiety, but also verbal or physical aggression, aberrant motor behaviour, and disinhibition can be interpreted as expressions of this suffering [7]. According to numerous studies, it can be assumed that, over the course of the disease, at least one of these symptoms will affect nearly all individuals with dementia [13, 14] Therefore, the most important goal of non-pharmacological intervention for PWSDs should be to reduce BPSD. Cohen-Mansfield’s [15] research group was able to show positive effects on agitated behavioural symptoms by identifying individuals’ needs and then treating them with specially tailored interventions.

Because behavioural symptoms cause tremendous distress in PWSDs, they consequently influence the quality of life (Qol) of these individuals. This means that in terms of research on quality of life, reducing BPSD also seems to be an important goal. The frequently discussed question here is: Which factors influence the Qol of people with dementia? O’Rourke and colleagues identified four factors that influence the Qol of people with dementia: relationships (together vs. alone), agency in life today (purposeful vs. aimless), wellness perspective (well vs. ill), and sense of place (located vs. unsettled) [16]. Hence, these factors should also be addressed by a non-pharmacological intervention for PWSDs.

In fact, more than 80% of the cognitively impaired individuals in nursing homes suffer from BPSD [17]. With regard to the perspective of nursing staff, this means that BPSD “occur very frequently in the everyday work of nursing staff and represent a far greater burden for them than cognitive impairments” [18] . Three quarters of all nursing staff have to deal with verbally conspicuous and physically restless behavior on a daily basis. Up to 40% of working time is spent on managing BPSD [19]. A total of 26.8% of nursing staff feel burdened by these symptoms [20]. Due to this work related stress factors, health care workers have the greatest incidences in days spent unable to work [21].

Many professional caregivers frequently request practicable activation programmes for PWSDs, as these individuals are often difficult to integrate into existing group settings [22]. Mostly, PWSDs are treated in short units of individual occupation, for example, by applying basal stimulation or by administering snoezelen, although the evidence that these treatments work has been inconclusive [23, 24]. Both are one-on-one interventions and do not fulfil the needs for participation and meaningful activities in a group, social interaction, and social exchange. Cohen-Mansfield [12] identified, among other things, social interaction and meaningful occupation as the most important unmet needs of PWSDs, as well as sensory stimulation. Sakamoto and colleagues [25] also found that interactive involvement is more promising than passive involvement.

This is exactly where MAKS comes in. Through the use of a group setting and multimodality, it is possible to satisfy essential basic human needs, such as the needs for participation, movement, feelings of success, and meaningful activity. By satisfying these elementary needs, BPSD are reduced. Thus MAKS as a psychosocial intervention, represents the instrument of choice for reducing BPSD. Overshott and Burns [8] also came to the conclusion that psychosocial interventions offer an alternative to drug therapy for BPSD with very few side effects.

The objective of this paper is to describe the study protocol of the cluster-randomised MAKS-s study (**MAKS** for people with **s**evere dementia) by following the evidence-based reporting guidelines of the SPIRIT Statement [26].

## Methods/design

### Aims and hypotheses

The main aim of the MAKS-s study, which began in July 2019, is to find out whether a non-pharmacological intervention adapted to the special needs of PWSDs can reduce the BPSD of this target group (primary target group) and consequently improve their Qol. In addition, we expect that the intervention will enable the maintenance of basal activities of daily living (ADLs). There is also the possibility that this improvement will provide some relief for the formal caregivers (secondary target group), which will be shown in a reduction in the number of days spent unable to work.

Research hypotheses:

Based on the primary outcome:

I. By participating in MAKS-s, the BPSD and Qol of PWSDs
  will be significantly better in the intervention group than in the control group.

Based on the secondary outcome:

II. By participating in MAKS-s, the everyday practical skills of PWSDs
  will be significantly better in the intervention group than in the control group.
III. In the care facilities in which MAKS-s is conducted, the dementia-related stress experience of the nursing and care staff (secondary target group) involved in the care of the participants will be significantly better in comparison with the nursing and care staff in the control facilities.
IV. The assumed positive trend described in Hypothesis 3 will lead to a relevant reduction in the number of days spent unable to work for this group of individuals.

### Study design and setting

A two-armed cluster-randomised, controlled, multicentre, prospective longitudinal study with a waitlist control group design will be conducted to test the abovementioned hypotheses.

The nursing homes are located in five areas (German federal state): Bavaria, Baden-Württemberg, Thuringia, Rhineland-Palatinate, and Saarland. All nursing homes (clusters) participating in the study will be randomly assigned to the intervention or control groups at baseline (through random selection) by our cooperation partner “Institut für Medizininformatik, Biometrie und Epidemiologie (IMBE)” at the Friedrich-Alexander University Erlangen-Nürnberg. The clusters will be allocated by applying a tree-step stratification process by using “protective versus non-protective ward”, “area”, and “nursing home size” in decreasing order to balance the structural parameters.

The contact people in each nursing home are trained by the study headquarters with respect to the study protocol, instruments, and screening procedure before randomisation. Informed consent from the legal representatives of the PWSDs and the nursing staff (i.e., trained nurses) and care staff (i.e., staff with no medical training) will also be obtained before randomisation. Nursing homes will be informed about their group assignment in written form by the study headquarters. In addition, the nursing homes in the intervention group will receive further training on the implementation of the MAKS-s intervention. This treatment will be performed through the 6-month intervention phase. The nursing homes in the control group will not carry out any specific treatment but will continue providing “care as usual”.

Because we are investigating a non-pharmacological intervention, not everybody participating in the study can be blinded. Due to the severity of dementia, we can assume that the PWSDs will be blind to the conditions of the study. The therapists performing the MAKS-s intervention cannot be blinded. The raters (nursing staff) will be “semi-blinded”. They will know about the group assignment but will have no knowledge of the intervention. The testers, who are trained students from the study headquarters, who will conduct the tests, will be completely blinded.

Six months after baseline, the care staff for the control group will receive the same training on how to implement the MAKS-s intervention as the care staff for the intervention group. After the 6-month intervention phase, the intervention and the control groups will be free to decide whether to continue or to begin the MAKS-s intervention, respectively.

The observation period will last 12 months, with a total of four measurement points (baseline, 2, 6, and 12-month follow ups).

The data will be collected by means of nursing home documents, psychometric tests, and external rating scales (see the “Measures” section). All procedures were approved by the Friedrich-Alexander University Erlangen-Nürnberg Ethics Committee. The external GKV Spitzenverband (the institution providing funding) is being regularly informed of the progress of the study and the milestones that have been achieved as set forth in the trial application. The SPIRIT participant timeline is presented in Table 1. When important protocol modifications are required, we will inform the Ethics Committee, the funding institution, the nursing homes, and the platform for the trial registry. The trial registration data are displayed in Table 2.

**Table 1:**
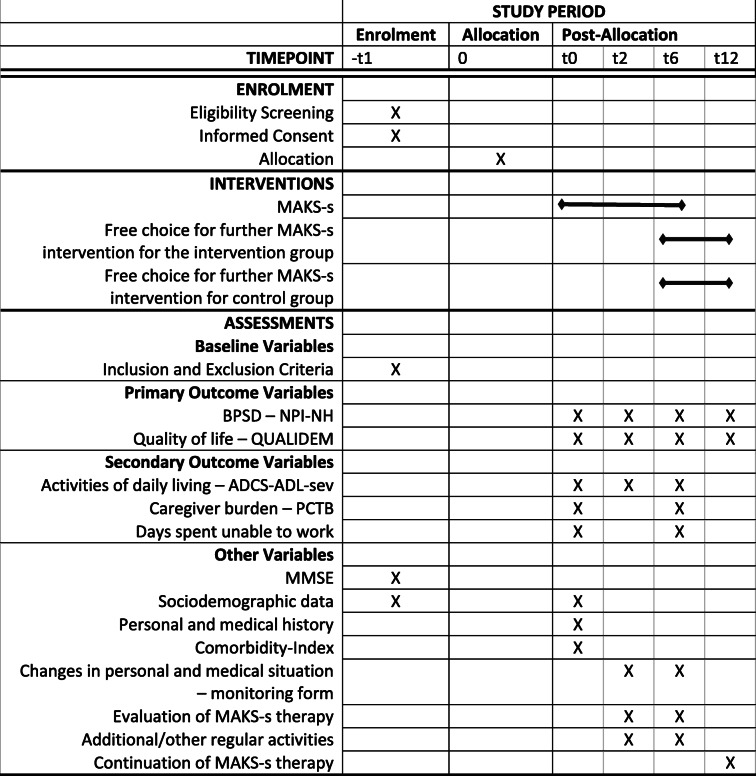
SPIRIT participant timeline

*Abbreviations*: *BPSD* Behavioural and psychological symptoms of dementia, *NPI-NH* Neuropsychiatric inventory nursing home version, *ADCS-ADL-sev* Alzheimer’s Disease Cooperative Study Activities of Daily Living scale for Severe impairment, *PCTB* Professional Care Team Burden (PCTB) Scale, *MMSE* Mini-Mental State Examination

**Table 2:** Trial Registration Data Set of the MAKS-s study

Data category	Information
Primary registry and trial identification number	ISRCTN15722923
Date of registration in primary registry	07/08/2019
Secondary identifying numbers	GKV-SV339
Source(s) of monetary or material support	German National Association of the Statutory Health Insurance and Long-Term Care Insurance Funds (GKV-Spitzenverband)
Primary sponsor	German National Association of the Statutory Health Insurance and Long-Term Care Insurance Funds (GKV-Spitzenverband)
Secondary sponsor(s)	–
Contact for public queries	Prof. Dr. Elmar Graessel (elmar.graessel@uk-erlangen.de)
Contact for scientific queries	Prof. Dr. Elmar Graessel (elmar.graessel@uk-erlangen.de)
Public title	MAKS-s – multimodal psychosocial intervention for people with severe dementia in inpatient care: a cluster-randomized controlled trial
Scientific title	MAKS-s – multimodal psychosocial intervention for people with severe dementia in inpatient care: a cluster-randomized controlled trial assessing the effects on behavioral and psychological symptoms, quality of life, and activities of daily living of people with severe dementia as well as caregiver burden
Countries of recruitment	Germany
Health condition(s) or problem(s) studied	severe dementia, professional care team burden
Intervention(s)	Intervention group: psychosocial group intervention Control group: treatment as usual
1.Key inclusion and exclusion criteria	Ages eligible for study: senior; Sexes eligible for study: all
	Inclusion criteria: 1. Psychometric verification of severe dementia syndrome: Mini Mental State Examination (MMSE) Score between 0 and 9 2. Informed consent for study participation.
	Exclusion criteria: 1. Mild to moderate dementia respectively no cognitive impairment (MMSE > 9) 2. Cognitive decline due to diseases other than dementia (e.g. schizophrenia or Korsakoff) 3. Severe hearing impairment 4. Severe visual impairment 5. Permanently bedridden persons 6. History of severe major depression 7. History of more than one stroke 8. No verbal communication in German possible
Study type	Cluster-randomized controlled multi-center intervention study
Date of first enrollment	01/12/2019
Target sample size	144
Recruitment status	ongoing
Primary outcome(s)	1. Quality of life, measured by QUALIDEM 2. Behavioural and psychological symptoms of dementia, measured by Neuropsychiatric Inventory Nursing Home Version (NPI-NH)
Key secondary outcomes	1. Activities of daily living measured by ADCS-ADL-severe 2. Dementia-related caregiver burden of the nursing and care staff, measured by Professional Care Team Burden Scale (PTCB) 3. Days of incapacity for work of nursing and care staff, measured by documentation of the employer 4. Extent of implementation and voluntary continuation of the intervention in follow-up period, measured by a self-developed questionnaire

### Sample size estimation

#### Primary target group

A power analysis was computed on the basis of the authors’ previous experience with the pilot study MAKS-s (Diehl, K: Modification and implementation of the MAKS-aktiv! therapy with severrly demented people: a pilot experiment, unpublished master´s thesis, Friedrich-Alexander University Erlangen-Nürnberg). For this purpose, the effects of the subscales “mood”, “disturbing behaviour”, and “social behaviour” of the NOSGER (“Nurses’ Observation Scale for Geriatric Patients”) [27] were pooled. The result was an effect size of Cohen’s *d* = 0.52. The beta-error was set to four times the size of the alpha error (α = .05), as proposed by Cohen [28]. This resulted in a power of .80.

Under these conditions, an evaluation sample of 114 individuals (57 per group, 6 per nursing-home) was deemed necessary. On the basis of the previous MAKS study [10], we expect a drop-out rate (mainly caused by death or being bed-ridden) of 20% across 6 months. This means a total of 144 people with severe dementia has to be included in the study. To achieve this goal, only nursing homes with at least 40 residents are being included in the project. This could be deduced from the following considerations:

- In nursing homes in Germany, 38.8% of residents suffer from severe dementia [1]. This means 15 out of 40 individuals.
- In the “MAKS aktiv” study, the rate of bedridden and/or blind/deaf residents was 26% [10]. In the group of severe dementia cases, this rate was 40%. This left 9 of the 15 people with severe dementia suitable for intervention.
- It can be assumed that at least 70% of the participants will be willing to participate after the legal representatives have been informed [10]. Thus, 6 of the 9 suitable PWSDs can be included in the study.

#### Secondary target group

The sample size of the secondary target group could not be deduced empirically. The number of members from the secondary target group will depend on the structural conditions of the nursing homes. In every nursing home, there must be a minimum of 6 individuals (MAKS-s therapists and other nursing staff) who meet the criteria for inclusion in the study.

### Recruitment strategies

Nursing homes from the postulated size in the recruitment areas are identified by means of their websites. Information material is sent to these nursing homes by post. Two weeks later, all the facilities that have been contacted are called by telephone and asked whether they are willing to participate. Cooperation contracts are signed with the nursing homes that are interested in participating in the study. After at least 24 nursing homes have signed a cooperation contract, recruitment will be completed.

### Eligibility of participants

All residents of the participating units in the 24 nursing homes will be included in the screening process. Additionally, in every nursing home, the members of the nursing and care staff who are involved most in the care of the participants of the study will be selected. The screening process will be conducted by trained nursing staff working in the participating nursing homes.

#### Primary target group

All residents will undergo a three-step standardised screening process to determine their suitability for the project. In the first step of the screening process, all residents fulfilling at least one of the following criteria are excluded: bedridden, blind, deaf, no sufficient knowledge of German, more than one stroke, severe depression, schizophrenia, an addictive disorder, concrete plan to leave the facility. In the second step, cognitive performance is assessed. Individuals who definitely have a MMSE (Mini-Mental State Examination) figure of over 9 points (estimated by the nursing staff) are excluded. Every other person is tested with the MMSE. Individuals who attain more than 9 points are excluded. All individuals who are potentially eligible to participate in the study are put on a list and randomised by a randomising list from our cooperation partner IMBE. After the randomisation, the nursing home staff apprise the legal representatives of the eligible individuals in the order of the randomisation list. After the legal representative provides written informed consent, the PWSD is included in the study. This process is performed until six people per nursing home give their consent.

#### Secondary target group

To select the individuals who are the most involved in the care of the participating PWSDs, three groups are formed. The first group consists of the therapists who will carry out the MAKS-s treatment. The second group is the nursing staff comprising the primary nurse and their stand in. The third group consists of the care staff involved in the occupational therapy of the study participants (in Germany, these are people who are trained to provide occupational therapy, but they are not nurses with degrees). The eligible nursing and care staff members are also informed about the study, and written informed consent is obtained from them.

### Intervention

The MAKS-s intervention is a multicomponent non-pharmacological intervention designed specifically for people with severe dementia. It consists of four components that are combined in the same order every day in a manualised intervention module lasting approximately 1 h. The participants have to use social skills in order to interact in a group. Social cooperation is promoted in all components.

#### The contents of the MAKS-s intervention

Table 3 provides an example of how the MAKS-s intervention is structured for 4 weeks. Due to the severe impairment of the PWSD, the 4-week plan can be repeated continuously. The daily treatment module begins with a social warm-up session, lasting approximately 10 min. Due to the amount of social contact in the group, the basic need for participation and social interaction is met. The social warm-up includes a welcome ritual, singing together, and some other recurring elements. The entire social warm-up is always conducted in the same order. Next is the sensorimotor session, which lasts about 20 min. The sensorimotor activation training meets the need for movement. After a motor ritual and a warm-up, exercises with handtools such as mini bean-bags or spiky massage balls are performed. Particular attention is paid to contracture prophylaxis and the training of motor skills that still remain as well as the promotion of body awareness. Subsequently, the cognitive session, lasting around 10 min, is performed. The cognitive stimulation conveys the feeling of success by addressing basic subconscious sensory memories. Due to cortical atrophia in severe dementia, higher cognitive processes cannot be addressed in PWSDs. Instead, rather unconscious processes such as priming or multisensory stimulation is used to activate any remaining long-term memory content, for example, well-known songs or poems. Those contents, despite the severity of the disease, can still be accessed via key stimuli (priming) because the corresponding regions in the cortex are less affected by Alzheimer’s Disease [29]. In particular, one area of the cortex that is less affected by Alzheimer’s dementia, the somatosensory cortex [29], which processes sensory impressions of tactile perception and depth sensitivity, is addressed by making people feel things with great tactile appeal. The last component performed in the MAKS-s intervention is the training in ADLs for about 20 min. Basic everyday activities such as putting lotion on one’s hands or cutting a piece of fruit are performed to promote gross and fine motor skills and especially procedural memory to maintain basic everyday skills. Thus, meaningful activity is experienced. (further examples are presented in Table 3, right column).

**Table 3:** MAKS-s 4-week-plan

MAKS®-s 4-week-plan	Social warm-up session 10 min.	sensorimotor activation training 20 min.	cognitive stimulation 10 min	Training in acvtivities of daily living 20 min.
Monday 1	fixed ritual	throwing balls, mini-bean-bag	singing songs	fidget quilt
Wednesday 1	fixed ritual	playing football, twin-rubberball stick	sensory training	spreading bread
Friday 1	fixed ritual	marching, (spiky massage)balls	completing proverbs, poems, rhyms or fairy tales	washing hands
Monday 2	fixed ritual	throwing balls, mini-bean-bag	singing songs	fidget quilt
Wednesday 2	fixed ritual	Playing football, twin-rubberball stick	sensory training	Cutting fruits and eating with fork
Friday 2	fixed ritual	marching, (spiky massage)balls	completing proverbs, poems, rhyms or fairy tales	pinning and screwing
Monday 3	fixed ritual	throwing balls, mini-bean-bag	singing songs	fidget quilt
Wednesday 3	fixed ritual	Playing football, twin-rubberball stick	sensory training	spreading bread
Friday 3	fixed ritual	marching, (spiky massage)balls	completing proverbs, poems, rhyms or fairy tales	Nipping clothespins on towels
Monday 4	fixed ritual	throwing balls, mini-bean-bag	singing songs	fidget quilt
Wednesday 4	fixed ritual	Playing football, twin-rubberball stick	sensory training	peeling and cutting potatoes/eggs, eating with fork
Friday 4	fixed ritual	marching, (spiky massage)balls	completing proverbs, poems, rhyms or fairy tales	Free choice of activity

#### Implementation of the MAKS-s intervention

The nursing homes in the intervention group will commit themselves to carrying out the MAKS-s intervention in accordance with the manual every Monday, Wednesday, and Friday for 6 months. All study participants will participate in the intervention every day. MAKS-s will be carried out by two trained MAKS-s therapists, who have received 2 days of training. All materials used in the intervention will be given to the nursing homes in the intervention group at baseline and will be given to the control groups after the 6-month intervention period. Staff members at the study headquarters will be available to answer questions by phone or e-mail on weekdays. The project will not exert any influence over the pharmacological treatment of the participants or their individual participation in other activities. However, all these influences will be documented for all study participants. Due to findings from previous randomised-controlled trials, serious adverse events are not expected. Therefore, no stopping guidelines are necessary [10, 11].

### Measures

#### Primary outcome measures

*Neuropsychiatric inventory nursing home version (NPI-NH)* [30]*.* The NPI-NH, a deduction from the original Neuropsychiatric Inventory (NPI) [31], is a comprehensive retrospective observer rating scale for assessing the BPSD of individuals who reside in nursing homes by interviewing their formal caregivers. The NPI-NH evaluates 10 behavioural areas (delusions, hallucinations, agitation, depression, anxiety, apathy, irritability, euphoria, disinhibition, aberrant motor behaviour) and two types of neurovegetative changes (night-time behaviour disorders and appetite and eating abnormalities). The severity and frequency of each symptom are assessed by means of structured questions administered to formal caregivers. Multiplying the values of severity (1–3) and frequency (1–4) results in the score of the respective symptom, ranging from 0 to 12. The total NPI score ranges from 0 to 144 [32]. In addition, the impact of behavioural disturbances on formal caregivers can be scored by summarizing the occupational disruptiveness. The German version showed a moderate internal consistency of .55 and .68 (Cronbach’s α) and a relatively robust factor structure [32] confirming the results of studies from other countries [33, 34].

*QUALIDEM* [35, 36]*.* To measure Qol, the German version of the QUALIDEM is used [37]. The QUALIDEM is a dementia-specific Qol instrument that allows a retrospective proxy-based rating, assessed by formal caregivers to be applied in a residential setting. QUALIDEM consists of two consecutive versions that can be used in the different stages of dementia [38]. The version we will use in this study was developed for people with very severe dementia. It contains 18 items, categorised into six domains of Qol: care relationship (3 items), positive affect (4 items), negative affect (2 items), restless or tense behaviour (3 items), social relations (3 items), and social isolation (3 items). The response options for all items are “never”, “extremely rare”, “rarely,” “sometimes”, “often”, “frequently”, and “very frequently”, thereby resulting in an item score that ranges from 0 to 6. Higher scores indicate a higher QoL. The scores on the subscales are calculated by adding up the item scores [38]. The global score is calculated by adding up and transforming the subscale scores into values that can range from 0 to 100 (theoretical range) according to Dichter et al. [39]. The scores on the subscales yield a Qol profile [38]. Studies on validity have provided evidence of satisfactory construct validity [35, 40]. The application of a user guide results in a sufficient interrater reliability for the QUALIDEM subscales [37].

#### Secondary outcome measures

##### Primary target group

*Alzheimer’s Disease Cooperative Study Activities of Daily Living scale for Severe impairment (ADCS-ADL-sev)* [41]*.* The ADCS-ADL-sev is a questionnaire that is provided as an interview to the caregiver of the PWSD. It is a retrospective observer rating scale relating on the capability to execute one specific ADL, assessing the preceding 4 weeks. Each of the 19 items consists of a series of hierarchical questions. The possible answers range from total independence to total inability. The total point score ranges from 0 to 54, with higher scores indicating higher ADL performance.

The items from the original ADCS-ADL [42] scale were individually validated. A subset of 19 items met the criteria for applicability, reliability, good scaling, concordant validity, and sensitivity to detect change in performance over 6–12 months in PWSDs [41].

##### Secondary target group

*Professional Care Team Burden (PCTB) Scale* [43]*.* The PCTB is a self-rating scale for assessing the subjective burden of the professional care staff in nursing homes and other caregiving facilities. The measure contains 10 items divided into 3 subscales (structural burden, objective burden, subjective burden). The items are rated on a five-point scale ranging from strongly disagree to strongly agree. The total score ranges from 0 to 40 points, with lower values indicating higher burden.

The convergent validity was demonstrated by computing the correlation with the Perceived Stress Scale (PSS), indicating moderate to high validity [43]. Cronbach’s alpha for the complete scale was .79.

*Days spent unable to work*. The number of days spent unable to work by the nursing and care staff who are most involved in the care of the participating PWSDs will be recorded by the head of the nursing home.

#### Other measures

*Mini-Mental State Examination (MMSE)* [44]*.* The MMSE is the most popular screening test for dementia [45]. It evaluates five domains of cognitive abilities: orientation, registration, attention and calculation, recall, and language. The score ranges from 0 to 30 points, and 0 to 9 points indicates severe dementia.

*Sociodemographic data, personal and medical history, and comorbidity index.* The following data on each participant’s medical history will be collected by the nursing home staff at baseline: sociodemographic data (age, sex), level of care, medications, and diagnosis. Comorbidities are weighted using the updated and validated Charlson Comorbidity Index (CCI) [46]. It is used to calculate the effect of 12 medical diagnoses on the mortality rate. Higher scores indicate a higher 1-year mortality rate. The 1-year mortality increases from 12% (index = 0) to 85% (index ≥5). Validity and excellent reliability have been demonstrated in several studies [47, 48].

*Monitoring form.* During the 6-month intervention period, every change in the health situation of the PWSD will be documented: change in the level of care, change in medication, additional diagnosis, hospitalisation, absences, death, and retirement from the MAKS-s intervention (intervention group only).

*Evaluation of MAKS-s.* The implementation of the MAKS-s intervention will be documented by the MAKS-s therapists every week. The attendance of every participant of the study will be recorded, along with deviations from the manual in order to monitor the intensity and the quality of the MAKS-s intervention.

*Additional/other regular activities.* All other activities, such as individual care, basal stimulation, singing, gymnastics, arts and crafts and so on, will be documented by the care staff every week.

*Continuation of the MAKS-s intervention.* To get indications of sustainability of MAKS-s, 12 months after baseline, all nursing homes participating in the study will be interviewed about the implementation of the intervention. They will be asked about the frequency, duration, number of participants, and whether it is practical to incorporate into the everyday routine.

### Data collection

All data will be collected at the nursing homes. The variables referring to PWSD will be collected by psychology students who are employed and trained by the study headquarters. To reduce reporting bias, the formal care staff providing data on the PWSDs (NPI-NH, QUALIDEM, ADCS-ADL-sev) will not be involved in the MAKS-s intervention. Beyond this, the data collectors (trained students) will be blinded. The variables pertaining to the secondary target group will be collected by the contact people in the nursing homes. All data will be collected in written form. These data will be stored in a locked steel cabinet, and only the researchers involved in the MAKS-s study will have access to the data.

A pseudonym will be created for every member of the primary target group (PWSDs), and it cannot be retraced. The people in the secondary target group will give themselves a pseudonym.

### Data quality management

All individuals involved in the study are thoroughly trained in their specific task. The interviewers/testers undergo a 2-day training course on how to administer the questionnaires and follow the data collection guidelines. The contact people in the nursing homes receive 3 h of teaching on the study protocol, and the MAKS-s therapists receive 2 days of training on how to implement the intervention. If questions arise, the study headquarters can be contacted by phone or e-mail.

100% of the data will be checked for the completeness and correctness of the screening criteria, as part of the managing of data entry. We will also verify that informed consent was obtained from all participants or their legal representatives.

In order to ensure the quality of the data, a minimum of 5% of the data will be subject to a random audit. To obtain evidence of the interrater reliability, 5% of the QUALIDEM, the NPI-NH, and ADCS-ADL-sev will be collected from two different raters. Two nursing homes in the intervention group (15%) will be visited by the staff from the study headquarters to observe how the MAKS-s intervention is being conducted (treatment adherence). At the same time, we will check for the quality of the documentation (deviations from the manual) and the regular attendance of the study participants. The data received by the study headquarters will also be carefully checked for plausibility and inconsistencies. The data will be assessed for completeness. The same procedure will be applied to the follow-up data. After all data have been collected, the ranges of the items and the relationships between the variables will be checked for their plausibility.

### Analysis

#### Data analysis

In order to revise the quality of randomisation, the baseline data from the intervention and the control groups will be compared for statistically significant differences.

Descriptive statistics will be reported on all variables from the primary target group (PWSDs), such as age, sex, level of care, or Charlson Index, and from the secondary target group (formal caregivers), such as age, sex, or profession.

Hypotheses 1 to 3 will be tested by applying multiple regression analyses. To ensure the robustness of the results, both data analytic strategies “intention to treat” and “per protocol” will be applied. “Intention to treat” evaluations are carried out with all cases are still alive at the end of the intervention period.

Differences in days spent unable to work among the formal caregivers in the intervention group facilities compared with those in the control group (Hypothesis 4) will be tested for significance using the *t*-test for independent groups.

#### Economic evaluation

To show the effects on the cost to the health care system, we will conduct an economic analysis of the days spent unable to work for the nursing and care staff members who are primarily involved in the care of the PWSDs.

Missing data will be imputed by using the expectation maximum (EM) algorithm. The data analysis will be performed with the “IBM SPSS Statistics 24” software. All analyses will be conducted under the supervision of the cooperation partner IMBE.

## Discussion

In this article, we described the design of a cluster-randomised controlled study on the effects of a multicomponent intervention for PWSD carried out in nursing homes. The effects of the intervention on the nursing home staff will also be investigated.

BPSD are the most challenging and the most burdensome side effects of this disease when it is in its severe stages. In addition to the formal caregivers, the PWSDs also suffer [17]. This indicates that the reduction of BPSD should be the main aim of a psychosocial intervention to reduce the strain of the formal caregivers and improve the quality of life of the PWSDs. The intervention described in this study is designed to reduce non-cognitive symptoms by providing a multicomponent intervention to meet the needs of the PWSDs.

### Strengths and limitations of the study design

The study design offers several strengths. The external validity of the study is high because of the naturalistic setting in nursing homes with nursing staff and the recruitment of nursing homes that are in different federal states of Germany, are in urban and rural regions, and are run by a wide variety of organisations (profit, non-profit, local authorities). Also the duration of the intervention of 6 months, the use of a manualised treatment, and the focus on two target groups (PWSDs and the nursing and care staff) have rarely been applied in international studies [15, 25, 49–51].

Another strength of the current study is the cluster randomisation. We decided to use cluster randomisation rather than randomisation because of the secondary target group. If we were to have a treatment group and a control group in the same nursing home, we could not observe any effect of the intervention on the formal caregivers because the caregivers may be responsible for both the people in the intervention group and the people in the control group. Furthermore, the intervention could not be withheld from any individual working in a nursing home without the possibility of “contaminating” the control group [52]. In order to avoid a systematic difference between the two arms of the study by chance, a three-step stratification process is being conducted to allocate the nursing homes. At the same time, to avoid a recruiting bias, recruitment will be completed before randomisation.

However, a limiting bias can occur. Because we are conducting a non-pharmacological intervention, the nurses who carry out the assessments (raters) are not completely blind to the conditions of the study because they know about the group assignment.

Because the study deals with PWSDs, due to the severe cognitive impairment of the participants, we could only use proxy-based ratings from an observer’s perspective. The perspective of the PWSDs themselves cannot be taken into account.

Because participating in the study requires extra work by the nursing home staff, the facilities of the control groups may be less motivated because they will not receive the intervention during the first 6 months. To motivate them, they will also receive the intervention at the end of the 6-month intervention period (waitlist control group). As the control group will also be trained in the MAKS-s intervention after a delay of 6 months, important indications for implementation can emerge.

## Data Availability

The research group intends to publish data generated from this study in open-access, peer-reviewed journals. The datasets used and/or analysed during the current study will be available from the corresponding author (elmar.graessel@uk-erlangen.de) on reasonable request.

## Abbreviations

ADLs: Activities of daily living
BPSD: Behavioural and psychological symptoms of dementia
MMSE: Mini-Mental State Examination
NPI-NH: Neuropsychiatric inventory nursing home version
PCTB: Professional Care Team Burden Scale
PWSD: Person with severe dementia
Qol: Quality of life
